# The Role of Toll-Like Receptors and Vitamin D in Cardiovascular Diseases—A Review

**DOI:** 10.3390/ijms18112252

**Published:** 2017-10-27

**Authors:** Daria M. Adamczak

**Affiliations:** Department of Cardiology, Poznan University of Medical Sciences, Dluga Street ½, 61-848 Poznan, Poland; daria.m.adamczak@gmail.com; Tel.: +48-61-854-9146

**Keywords:** toll-like receptors, vitamin D, cardiovascular diseases, atherosclerosis, inflammation

## Abstract

Cardiovascular diseases are the leading cause of mortality worldwide. Therefore, a better understanding of their pathomechanisms and the subsequent implementation of optimal prophylactic and therapeutic strategies are of utmost importance. A growing body of evidence states that low-grade inflammation is a common feature for most of the cardiovascular diseases in which the contributing factors are the activation of toll-like receptors (TLRs) and vitamin D deficiency. In this article, available data concerning the association of cardiovascular diseases with TLRs and vitamin D status are reviewed, followed by a discussion of new possible approaches to cardiovascular disease management.

## 1. Toll-Like Receptors

Toll-like receptors (TLRs) are members of the pattern-recognition receptors (PRRs) family and play a key role in the phylogenetically ancient innate immune system. Due to PRRs, the eukaryotic organism is able to discriminate between self and non-self antigens [1,2,3]. There are 13 TLRs in mammalian species, and each one perceives a specific ligand, with the exception of TLR11 that is not functional, while TLR12 and -13 are not expressed in humans [4]. TLRs are cell surface and intracellular (TLR3, -7, -8 and -9) single, membrane-spanning, non-catalytic receptors expressed mainly on sentinel cells such as macrophages and dendritic cells. Their name derives from homology to the *Drosophila* Toll molecule—an important component of dorsal-ventral patterning and antifungal defense.

TLRs recognize conserved microbial structures called PAMPs (pathogen-associated molecular patterns) as well as host biomolecules associated with cell damage or necrosis called DAMPs (danger-associated molecular patterns) and induce an immune response. They utilize leucine-rich-repeat motifs (similar to interleukin (IL)-1) to bind the ligands and a shared cytoplasmic domain to recruit the following adaptors for downstream signaling: MyD88 (myeloid differentiation factor 88), TRIF (TIR-domain-containing adaptor inducing interferon-β), TIRAP (TIR domain-containing adaptor protein) and/or TRAM (TRIF-related adaptor molecule) [5,6]. The crucial end-point of the cascade reactions is unmasking the nuclear localization domain of NF-κB (nuclear factor κ-light-chain-enhancer of activated B cells), which after its translocation into the nucleus, activates multiple pro-inflammatory genes [7,8]. TLR stimulation via PAMPs (e.g., microbial nucleic acids, bacterial lipoglycans, carbohydrates and peptides, protozoan glycosylphosphatidylinositol anchors, fungal glucans and chitin) or DAMPs (e.g., heat-shock proteins, HMGB1 (high-mobility group box 1), uric acid, ATP and DNA) leads to synthesis of type I interferons via the TRIF-dependent pathway and pro-inflammatory cytokines via the MyD88-dependent pathway, as well as the maturation of dendritic cells and even in some cases an induction of the adaptive immune system [9,10,11]. Additionally, TLRs play a role in the regulation of immune responses (suppression and contrasupression) by direct and indirect influence on the function of CD4^+^ CD25^+^ (cluster of differentiation) T regulatory cells (Tregs).

Finally, mounting evidence demonstrates that TLRs and the innate immune system are involved in the pathogenesis of disorders associated with chronic inflammation such as diabetes mellitus, asthma, Crohn’s disease, systemic lupus erythematosus, cancer and cardiovascular diseases, which suggests new possible therapeutic targets [12,13].

### 1.1. Toll-Like Receptors and Cardiovascular Diseases

Apart from being expressed in immune cells, TLRs are expressed in other cells found in the epithelium, endothelium, as well as adipocytes and those of the cardiovascular system [1,14,15,16]. Messenger RNA for TLR1–10 has been detected in the human heart [17]. Nevertheless, the role of the innate immune system in the pathogenesis of cardiovascular diseases has been discovered only recently. The most investigated receptor in this area is TLR4. Aggregated data suggest that short-term activation of TLRs has a cytoprotective effect on the cardiovascular system, whereas prolonged or excessive activation of TLRs induces chronic low-grade inflammation, which leads to endothelial dysfunction, increased cell death, adverse cardiac remodeling and subsequently coronary and cerebrovascular atherosclerosis, heart failure, septic cardiomyopathy, viral myocarditis, valvular diseases, thrombosis and/or hypertension [4,11,14,15,18,19]. Furthermore, cardiovascular risk factors such as diabetes, obesity and insulin resistance, are also associated with a low-grade inflammation that mimics the activation of innate immunity associated with metabolic, environmental, and genetic factors [20]. Taking into account that cardiovascular diseases are the leading cause of mortality worldwide (17.6 million deaths in 2016 with 14.5% rise from 2006 to 2016), thorough knowledge of the pathomechanisms of TLRs is very important [21].

### 1.2. Coronary and Cerebrovascular Atherosclerosis

There is a growing body of evidence that innate immune mechanisms may initiate and accelerate atherosclerosis [22]. Normal arteries show very low levels of all TLRs, with the exception of TLR4, found at a relatively higher level [23]. However, in atherosclerotic lesions, TLR1, -2, -4 and -5 have been identified in abundance. It is important to note that TLR4 is concentrated and upregulated (likely by oxidized low-density lipoproteins) in areas of atherosclerotic plaques most vulnerable to rupture. TLR4 also contributes to intimal foam cell accumulation as well as TLR2, although to a lesser extent. Additionally, TLR signaling induces leukocyte recruitment and enhances matrix metalloproteinase expression within atherosclerotic lesions, therefore weakening plaque caps [4,24,25,26,27]. Furthermore, hyaluronic acid, a constituent of the extracellular matrix of eroded lesions, serves as a DAMP of TLR2, which is involved in promoting endothelial cell apoptosis [28,29]. Studies from the early 2000s emphasized the key role of MyD88 in atherosclerosis. It was hypothesized that its inactivation led to reduced macrophage recruitment to the artery wall and diminished plaque formation. However, recent data suggest that the impact of MyD88 on CD11c^+^ dendritic cells (CD11c^+^ DCs) is significant. CD11 is a transmembrane protein found on immune cells, which induces cellular activation and helps trigger neutrophil oxidative burst. The dominant effect of MyD88 signaling in CD11c^+^ DCs is to promote their maturation and the development of atheroprotective Tregs as well as proatherogenic T effector (Teff) cells. Tregs exert their atheroprotective action via suppression of Teff cells and inflammatory macrophages, and they inhibit monocyte recruitment by decreasing MCP-1 (monocyte chemotactic protein-1) production, a proces dependent on TGF-β (transforming growth factor β) [30,31,32]. However, one study revealed that in the absence of MyD88 signaling in CD11c^+^ DCs, the loss of Tregs response surpassed the loss of T effector cell activation [33]. On the contrary, another study showed that fewer Tregs are found in atherogenic *ApoE*^−/−^ (apolipoprotein E knockout) mice comparing to wild-type controls, suggesting an imbalance between Tregs and Teffs in atherogenesis. In addition, MyD88 signaling in CD4^+^ T cells is required to overcome suppression by Tregs. Moreover, CD4^+^ T cell anergy generates Tregs precursors [34,35,36]. Based on this evidence, it can be concluded that the impact of TLRs on atherosclerosis is a much more complicated issue than previously thought. Furthermore, TLR3 that signals via TRIF and is MyD88-independent unexpectedly plays a protective role in plaque formation [37]. It may stem from the ability of TLR3 to induce the expression of cytoprotective and anti-inflammatory glycoprotein clusterin/apolipoprotein J to prevent the progression of atherosclerosis [38].

### 1.3. Acute Coronary Syndrome

Besides promoting atherosclerosis, TLR-induced inflammation may influence atherosclerotic plaque stability and contribute to the development of acute coronary syndromes in patients with coronary artery disease. Microbial products within the plaques may promote its growth and rupture by activating inflammatory cells [39]. Recent studies revealed that median TLR2 and -4 expressions on platelets and plaque debris were greater in patients with acute coronary syndrome compared to those with stable angina pectoris [40,41]. Smaller infarctions and less inflammation has also been observed in mice subjected to myocardial ischemia-reperfusion and treated with TLR2 or TLR4 antagonists (anti-TLR2 antibody and Eritoran, respectively) [42,43]. In addition, the same effect occurred in TLR4-deficient mice [44]. Increased activation of TLR2 and -4 was observed also in human circulating monocytes during acute coronary syndrome. The extent of TLR activation preceded massive troponin T release and correlated with the ischemic area measured by cardiac magnetic resonance. On the other hand, TLR4 signaling plays a potent role in cardiac protection against ischemia-reperfusion injury via inducible nitric oxide synthase (iNOS) [45].

### 1.4. Stroke

Brain damage caused by stroke may be exacerbated due to acute inflammation triggered by cerebral ischemia [46]. The regulation of post-stroke inflammation involves vascular effects, chemotaxis, cellular responses and necrosis [47]. TLRs are expressed on endogenous neural cells along with infiltrating immune cells that access lesions due to damaged blood-brain barrier. The role of TLRs in cerebrovascular injury can be classified into two categories. The first category is post-ischemic TLR activation that mediates neuroinflammation and neurodegeneration (TLR2, -4, -5, -7, -8, and -9). The second category is pre-ischemic TLR stimulation, which is neuroprotective and preconditions brain to oxygen and nutrient deprivation (TLR2, -3, -4, -7, and -9) [48,49,50,51,52,53,54]. Recent studies revealed that TLR2- and TLR4-deficient mice had significantly less neurologic deficit following induced cerebral ischemia compared to controls [55,56]. On the other hand, preconditioning with a TLR2 ligand protected the brain from ischemia-reperfusion injury, possibly through a TLR2/PI3K/protein kinase B-dependent mechanism [57,58]. Additionally, treatment with TLR4 antagonist reduced neuroinflammation and neurologic deficits after intracerebral hemorrhage [59]. Surprisingly, TLR4-deficient mice exhibited worse outcomes versus controls after preconditioning [60]. The explanation of this phenomenon may be the ability of TLR4 to promote neurogenesis after stroke [61].

### 1.5. Thrombosis

Thrombosis is the formation of a blood clot inside a blood vessel, which leads to ischemia of the obstructed area. Platelets play a key role in this process. Furthermore, all 10 TLRs are found in human thrombocytes and their activation leads to platelet adhesion, aggregation, and production of mixed platelet-leukocyte formations, secretion of cytokines and chemokines, as well as reactive oxygen species (ROS) and thrombin generation [62,63]. Therefore, activation of the innate immune system, either by pathogens or by tissue damage, can lead to thrombosis and consecutive coronary events.

### 1.6. Heart Failure

Congestive heart failure may occur due to many pathological conditions such as coronary artery disease, hypertension or valvular dysfunction. The compensatory, but otherwise maladaptive remodeling process following a myocardial infarction leads to diastolic impairment and further deterioration of cardiac function. TLRs are involved in heart failure pathogenesis and myocardial remodeling [19]. It has been proven that TLR4 expression is increased in patients with advanced heart failure [64]. TLR2, -4, -5 and -9 signaling results in a strong inflammatory response via NF-κB and decreased contractility in human and murine cardiomyocytes [65,66,67]. TLR4 triggers not only an inflammatory response, but also extracellular matrix degradation, which contributes to left ventricular remodeling. Its deficiency has an opposite effect, at least in murine models [68].

### 1.7. Valvular Heart Disease

The role of TLRs in valvular disease is not very well known, however a few studies revealed a link between innate immunity and aortic valve calcification. TLR2 and -4 increase gene expression of osteogenic factors, such as BMP-2 (bone morphogenetic protein-2) and Runx2 (Runt-related transcription factor 2) [69,70]. Although more research is needed to fully understand the role of TLRs in valvular heart disease, a small study showed that plasma and tissue TNF-*α* (tumor necrosis factor alpha) and IL-6 (interleukin 6) levels correlated with the calcification of human aortic valve, which may be a result of TLR2 and -4 upregulation [71].

### 1.8. Arrhythmias

Arrhythmias are another serious cardiovascular problem affecting many people worldwide with increasing mortality. The latest data suggest that TLR4 activation promotes cardiac arrhythmias by decreasing the transient outward potassium current (Ito) through an interferon regulatory factor 3 (IFN3)-dependent and MyD88-independent pathway [72]. Furthermore, research in mice models revealed that TLR2 signaling triggers fatal arrhythmias upon myocardial ischemia-reperfusion. Absence of TLR2 improved survival toward 100% [73].

### 1.9. Hypertension

The pathogenesis of hypertension is a complex issue, considering the involvement of many factors such as the kidneys, autonomic nervous system and arteries as well as the uncontrolled activation of immune system and inflammation. However, TLRs have been suggested as the unifying factors in these processes [74,75]. TLR4 contributes to the etiology of vascular dysfunction and hypertension in various experimental models such as a spontaneously hypertensive rat (SHR) and rats with angiotensin II-induced hypertension [76,77]. Some authors even recognize angiotensin II as a DAMP for TLR4, as well as for TLR7–9 [78,79]. TLR4 also plays a role in paraventricular nucleus-mediated autonomic dysfunction in those animal models [80,81]. On the other hand, TLR5 has not been directly linked with high blood pressure, but it contributes greatly to metabolic syndrome [82]. Furthermore, TLR9 inhibition in SHR led to a decrease in blood pressure, whereas its activation induced vascular dysfunction and increased blood pressure in normotensive rats [79,83]. Finally, it was observed that TLR9 is a negative regulator of cardiac vagal tone and baroreflex function [84,85].

### 1.10. Cardiac Involvement in Infectious Diseases

Although further research is required to determine the full spectrum of endogenous molecules leading to myocardial damage in sepsis, recent studies have shown that mice deficient in TLR4 or IRAK1 (interleukin-1 receptor-associated kinase 1) are protected from mortality induced by lipopolysaccharide (LPS) and cardiac dysfunction [85,86]. Activation of TLR4 by LPS results in production of TNF (tumor necrosis factor), interleukin 1β (IL-1β), NO (nitric oxide) and NOS2 (nitric oxide synthase 2) [87]. Additionally, TLR2 and -9 knockout mice are protected from *Staphylococcus aureus* and synthetic bacterial DNA, respectively, thus proving multiple TLRs are involved in septic cardiomyopathy pathogenesis [67,88].

Viral infections can activate innate immune signaling within the heart through Myd88-dependent (TLR7–9) and MyD88-independent pathways (TLR3). In TLR3 knockout mice infected with encephalomyocarditis virus (EMCV) and Coxsackievirus group B serotype 3 (CVB3), an earlier mortality was seen when compared to wild-type mice. This has been associated with increased viral replication and myocardial injury [89,90]. These results suggest that TLR3-mediated recognition of a viral infection, with subsequent activation of antiviral mechanisms (e.g., type I interferon response) may be crucial to minimize viral replication in the heart. Similar outcomes were seen in TLR9 deficient and MyD88 deficient mice infected with cytomegalovirus (CMV) [91]. On the other hand, studies performed in CVB3 infected MyD88 deficient mice showed improved survival in the study group when compared to the control one. Moreover, pathological examination of the hearts revealed a significant decrease in CVB3 titers and cardiac inflammation in those mice [92]. The discrepancy regarding the abovementioned findings is hard to explain, but may be related to the MyD88-independent antiviral mechanisms that have not yet been discovered. Nevertheless, the reviewed data suggest that TLR-mediated viral mechanisms in the heart play an important role in the pathogenesis of myocarditis.

## 2. Vitamin D

Vitamin D is a group of fat-soluble secosteroids. The most important compounds in humans are vitamin D_3_ (cholecarciferol) and vitamin D_2_ (ergocalciferol). They play an essential role in calcium homeostasis and bone metabolism. Vitamin D_2_ and D_3_ can be ingested from diet, although only few foods, such as fatty fish liver and egg yolks contain them. However, the major natural source of vitamin D_3_ is dermal synthesis from sun exposure, especially UVB radiation [93,94]. Vitamin D itself is a prohormone, that is enzymatically converted in the liver to the major circulating form 25-hydroxyvitamin D (25(OH)D; calcidiol). Afterwards, the resultant compound is transformed in the renal proximal tubules (and at least 10 other tissues) to the most active form 1,25-dihydroxyvitamin D (1,25(OH)(2)D; calcitriol). 25-hydroxyvitamin D is less than 1% as potent as 1,25-dihydroxyvitamin D, but, on the other hand, it has a half-life of 2–3 weeks compared with 4–6 hours for its active form. Therefore, 25(OH)D is used as a maker of vitamin D status in the organism. 1,25(OH)(2)D activates its cellular receptor VDR (vitamin D receptor), a member of the superfamily of hormone-activated nuclear receptors regulating eukaryotic gene expression. VDR acts as a transcription factor that binds to specific DNA sequences, HREs (hormone response elements), in target gene promoters [95]. It is nearly universally expressed in all nucleated cells and approximately 3% of the human genome is under the control of 1,25(OH)(2)D [96].

Apart from the well-known role of vitamin D, research during the past three decades has revealed a diverse range of its biological actions such as induction of cell differentiation, inhibition of cell growth, immunomodulation, and control of other hormonal systems [94]. The importance of vitamin D in extraskeletal health is evident due to the diverse pathology associated with vitamin D deficiency. There are two conflicting statements concerning vitamin D optimal status. The first one requires serum 25(OH)D at 20 ng/mL, at least, to regulate calcium and bone homeostasis. The second one demands higher concentrations to maintain extraskeletal health. Some authors favor levels 20–40 ng/mL (50 to 100 nmol/L), whereas other experts support levels 30–50 ng/mL (75 to 125 nmol/L), since concentrations >150 ng/mL are associated with hypercalcemia and hyperphosphatemia [97,98,99,100]. Vitamin D deficiency is a major public health problem worldwide in all age groups, especially in infants, children and adolescents. In the National Health and Nutrition Examination Survey (NHANES) 2005 to 2006, 41.6% of adult participants (≥20 years) had 25(OH)D levels <20 ng/mL. Surprisingly, low vitamin D levels are a serious issue even in sunny regions, particularly in the Middle East [101,102,103]. Vitamin D deficiency is linked to not only rickets and osteoporosis, but also cancer, infectious, autoimmune, respiratory and finally cardiovascular diseases [104,105].

### 2.1. Vitamin D and Cardiovascular Diseases

A number of observational studies reported an inverse association between 25(OH)D levels and risk of cardiovascular diseases [106,107,108]. Vitamin D deficiency contributes to the development of cardiovascular diseases both directly and indirectly. VDRs are expressed in many tissues, including cardiomyocytes, vascular smooth muscle cells and endothelium. Calcitriol regulates the RAAS (renin-angiotensin system), suppresses vascular smooth muscle cells proliferation, improves insulin resistance and endothelial cell-dependent vasodilation, whilst also inhibiting myocardial cell hypertrophy, exertion of anticoagulant along with antifibrotic activity and finally modulates macrophage activity and cytokine generation [109]. Moreover, low levels of 25(OH)D are associated with cardiovascular risk factors, such as diabetes, obesity and dyslipidemia [110]. Elderly people are at risk for vitamin D deficiency as a result of decreased cutaneous synthesis and dietary intake. Epidemiologic studies indicate an association between low levels of vitamin D and diseases associated with aging, e.g., cardiovascular diseases [111]. Since screening and treatment of vitamin D deficiency are relatively easy, research concerning its connection to cardiovascular diseases, has important implications for patient care, as well as health policy [112]. However, there is a discrepancy between findings from observational studies and randomized trials on association between vitamin D and risk of cardiovascular diseases. Nevertheless, data from randomized controlled trials are characterized by a high degree of heterogeneity concerning optimal 25(OH)D concentrations, and only few of them specifically investigated effects in a population with low vitamin D levels [113,114].

### 2.2. Coronary and Cerebrovascular Atherosclerosis

Vitamin D deficiency is associated with endothelial dysfunction, inflammation, and vascular calcification—phenomena involved in pathogenesis of atherosclerosis [115,116,117]. The endothelial cells express VDRs and have an ability to synthesize calcitriol [118]. That strengthens the hypothesis that vitamin D modulates the endothelial function on autocrine and intracrine processes [119]. The protective effects of vitamin D on endothelium are exerted through genomic and nongenomic mechanisms. It suppresses the NADPH (reduced nicotinamide adenine dinucleotide phosphate) oxidase subunit p22(phox) and is a direct transcriptional regulator of endothelial NO synthase (eNOS), therefore increasing NO production, whilst decreasing ROS production [120,121,122]. VDR mutant mice are characterized by a lower bioavailability of the vasodilator nitric oxide, which leads to endothelial dysfunction, increased arterial stiffness, increased aortic impedance, structural remodeling of the aorta, and subsequently impaired systolic and diastolic heart function [123]. Calcitirol inhibits also the expression of interleukins 6 and 8, as well as RANTES (regulated on activation, normal T cell expressed, and secreted), ICAM-1 (intercellular adhesion molecule-1), PECAM-1 (platelet-endothelial cell adhesion molecule-1), VCAM-1 (vascular cell adhesion molecule-1), RAGE (receptor of advanced glycation end products) and E-selectin through NF-κB-mediated pathway [124,125,126,127,128]. Furthermore, vitamin D reduces the levels of prostaglandins by repressing the expression of COX-2 (cyclooxygenase-2) and upregulation the expression of an enzyme initiating prostaglandin catabolism, namely 15PGDH (15-hydroxyprostaglandin dehydrogenase) [129,130]. Finally, calcitriol reduces calcium influx into the endothelial cells and hence decreases the production of EDCFs (endothelium-derived contracting factors). It also directly downregulates the expression of COX-1 (cyclooxygenase-1), which is a major source of EDCFs [131,132,133]. Another interesting study revealed that oral administration of calcitirol decreases atherosclerosis in mice by inducing Tregs and immature dendritic cells with tolerogenic functions [134,135]. On the other hand, some researchers claim that prolonged or high dose vitamin D supplementation, unless needed to correct the deficiency, may even be immunologically harmful by downregulating Th1 immune responses and indirectly upregulating Th2 immune activation with potential disadvantageous metabolic and cardiovascular effects [136]. Nevertheless, patients with coronary artery disease and lower vitamin D concentrations tend to have higher troponin I levels [137].

### 2.3. Acute Coronary Syndrome

Vitamin D deficiency was also observed in acute coronary syndromes and was associated with a less favorable outcome. In a study of patients with myocardial infarctions, those who were in the lowest quartile of 25(OH)D levels had a higher risk for several in-hospital complications, including death, as well as one-year mortality. There were no observed differences in 25(OH)D concentrations between patients with ST-elevation myocardial infarction (STEMI) and non-ST-elevation myocardial infarction (NSTEMI) [138]. Another study supported those observations and indicated that vitamin D was independently related to mortality at levels below the population median [139]. In addition, the left ventricular function was more severely compromised in deficient patients [140]. Furthermore, the diabetic patients with 25(OH)D deficiency had more extensive coronary lesions [141]. From the molecular point of view, in vitro experiments in human endothelial cells showed that calcitriol decreased the expression of membrane type 1 matrix metalloproteinase and platelet activation through a reduction of the CD62p platelet adhesion molecule what may have therapeutic effects against destabilization of atherosclerotic plaques and thrombosis [128].

### 2.4. Stroke

Epidemiological studies have largely, however inconsistently, shown that vitamin D deficiency is a risk factor for strokes. It exerts neuroprotective, neuromuscular and osteoprotective effects which may reduce cognitive and functional impairments in post-stroke patients [142]. The latest research revealed that patients with ischemic stroke and vitamin D deficiency who received a single dose of 6 lac IU of Cholecalciferol Intramascular (IM) injection, had a significant improvement in the stroke outcome after three months of follow-up (assessed by Scandinavian Stroke Scale) compared to the control group [143].

### 2.5. Thrombosis

Although data concerning the relationship between vitamin D and thrombosis are very scarce, there is some evidence of low 25(OH)D levels associated with venous thromboembolism [144,145].

### 2.6. Heart Failure

Heart failure is associated with upregulation of the RAAS and sympathetic nervous system, which leads to alterations in β-adrenergic signaling, impaired mobilization of intracellular calcium and subsequently cardiomyocyte fibrosis, hypertrophy and interstitial collagen accumulation. As a result, negative ventricular remodeling occurs [146,147,148]. Available data indicate that the majority of congestive heart failure (CHF) patients have vitamin D deficiency [149]. Factors predisposing those patients to hypovitaminosis D include nutritional deficiency, decreased skin production, reduced intestinal absorption and hepatorenal disease [150]. Low vitamin D levels can in turn exacerbate CHF and lead to secondary hyperparathyroidism, which independently contributes to abnormalities in calcium homeostasis and subsequently left ventricular hypertrophy and diastolic dysfunction [151]. On the other hand, calcitriol decreases inflammation and downregulates pro-fibrotic signaling pathways by reducing TGF-β1 levels, stress fiber formation, and α-smooth muscle actin expression [152,153,154]. Moreover, patients with heart failure, who supplemented vitamin D, had higher serum concentrations of the anti-inflammatory cytokine IL-10 than the control group [155]. Some authors even postulate, that vitamin D-dependent mechanisms activating calcium channels may become a novel target therapy in heart failure patients [156]. What is interesting, in a population-based sample of mainly vitamin D-sufficient subjects without heart disease, the geometry of left ventricle was most favorable at intermediate 25(OH)D concentrations, namely 30–37 ng/mL [157].

### 2.7. Valvular Heart Disease

There are only few observational data concerning the relationship between hypovitaminosis D and valvular heart disease. On the other hand, aortic stenosis is the most common valvular heart disease in the developed countries and affects 2% people above 65 years old, while another 25–30% have aortic sclerosis. Since the mortality rate at 10 years in symptomatic patients is 90%, it is a serious medical problem [158,159,160]. Although there is currently no medical therapy that can prevent the progression of aortic stenosis, recent data highlight its possible association with bone metabolism. In patients with normal vitamin D levels, progression of the disease is associated with bone resorptive balance, whereas in patients with hypovitaminosis D secondary hyperparathyroidism plays a role [161]. Furthermore, in calcific aortic valve disease, the early lesion is similar to atherosclerotic plaque, but later calcification prevails. In patients with coronary artery disease, higher serum iPTH (intact parathyroid hormone) with lower vitamin D levels are independently correlated with calcific aortic stenosis [162]. Another study revealed a link between vitamin D deficiency, secondary parathyroidism, oxidative stress and aortic as well as mitral valve regurgitation [163].

### 2.8. Arrhythmias

In the pathogenesis of atrial fibrillation (AF), intra- and interatrial electromechanical delay, abnormalities in left atrial mechanical function, as well as myocardial fibrosis all play a role. Since vitamin D downregulates the production of ROS and activity of RAAS, its beneficial impact on AF has been hypothesized. However, the mechanistic and observational studies revealed conflicting evidence. Most of the studies, including the Rotterdam Study of 3.395 participants conducted for 12 years, do not support association of vitamin D with AF. Another research study revealed that hypovitaminosis D modestly increases the risk of AF [164,165,166]. In patients after coronary artery bypass surgery (CABG), the relationship between POAF (postoperative atrial fibrillation) with vitamin D deficiency is even more contradictory, although the significant negative correlation between 25(OH)D levels and left atrial diameter has been observed [167,168]. On the other hand, low plasma concentration of vitamin D was strongly associated with AF in patients with chronic CHF [169].

Although AF was the most investigated arrhythmia in context of vitamin D deficiency, the effectiveness of β-blocker treatment for premature ventricular complexes (PVCs) in patients with chronic kidney disease, was higher in subjects with a normal range of 25(OH)D in comparison to patients with deficiency or insufficiency [170].

### 2.9. Hypertension

The antihypertensive effects of vitamin D include suppression of RAAS, decreasing PTH (parathyroid hormone) levels, increasing urinary sodium excretion, as well as renoprotective, anti-inflammatory and vasculoprotective properties. Disruption of the VDR gene leads to an elevated renin production, left ventricular hypertrophy, and elevated blood pressure. Therefore, vitamin D deficiency was proposed as an independent risk factor for idiopathic arterial hypertension. Moreover, meta-analyses of randomized controlled trials showed that supplementation reduces systolic blood pressure by 2–6 mmHg [171,172,173,174,175,176]. Another meta-analysis of eight unique prospective cohorts (aggregated data of 283.537 subjects and 55.816 hypertension cases) has shown 30% reduced risk of hypertension with increasing vitamin D levels [177]. National Health and Nutrition Examination Survey (NHANES III) and Nurses’ Health study II were also favorable for vitamin D [178,179]. On the other hand, the largest randomized trial evaluating the effect of vitamin D supplementation on hypertension—the Women’s Health Initiative Calcium/vitamin D Trial of 36.282 postmenopausal women, along with Multi-Ethnic Study of Atherosclerosis Trial did not support the beneficial effect of vitamin D [180,181].

### 2.10. Cardiac Involvement in Infectious Diseases

Patients with sepsis have decreased vitamin D binding-protein (DBP) levels, which exacerbate the vitamin D deficiency. Furthermore, hypovitaminosis D is associated with increased risk of blood culture positivity [182]. The latest evidence has shown also that treatment of severely vitamin D-deficient, critically ill patients in the intensive care unit, who then receive high doses of vitamin D, may improve mortality [183]. In animal models of sepsis associated with a disseminated intravascular coagulation, administration of calcitriol resulted in improvement of blood coagulation parameters. In vitro studies revealed also that vitamin D modulates levels of the systemic inflammatory cytokines, such as TNF-α and IL-6, as well as inhibits the LPS-induced activation and vasodilation of vascular endothelium. It also enhances the induction of the antimicrobial peptides on mucosal and epithelial surfaces—β-defensin and cathelicidin—which have been described as the first line of defense against bacterial and viral pathogens [184]. Another study concerning mice injected intraperitoneally with Coxsackievirus B3 revealed significantly increased expression of VDR in myocardium in the experimental group. Moreover, the changes in the pathological score of the myocardium positively correlated with the changes in the expression of VDR. However, administration of vitamin D in those mice as a next step of the study, could reveal a clinical value of calcitriol [185].

## 3. Toll-Like Receptors and Vitamin D

### 3.1. The Interplay between TLRs and Vitamin D in Relation to Innate and Adaptive Immune System

**Figure 1:**
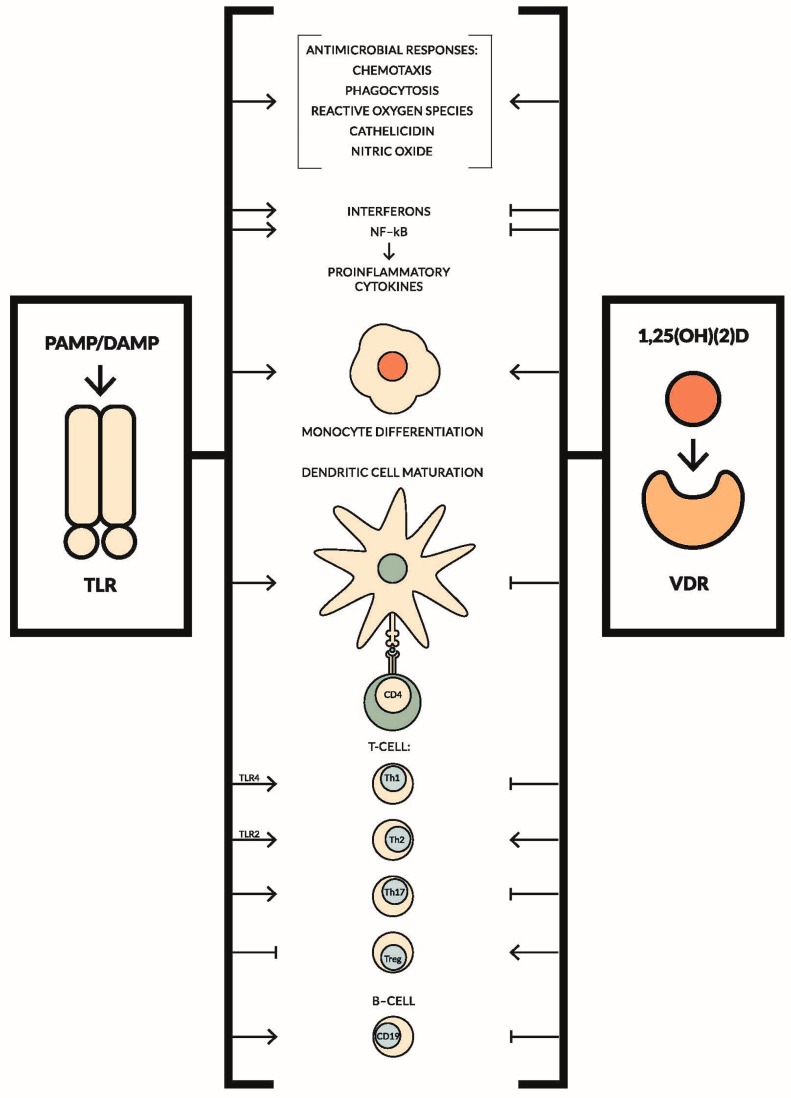
The role of toll-like receptors and vitamin D in innate and adaptive immune responses.

The effects of TLRs and vitamin D on the immune system are contradictory in most cases. Data concerning their impact on the innate and adaptive immune system are summarized in Figure 1 [186,187,188,189,190,191,192,193,194,195,196,197,198]. Although the effects of TLRs and vitamin D seem to be antagonistic, they produce synergistic effects during infectious diseases. On the other hand, a negative correlation between TLRs expression and 25(OH)D levels has been observed in many studies, especially in the diseases where inflammation plays a pivotal role, for example in diabetes mellitus type 1 or Behçet’s disease. Furthermore, both vitamin D supplementation, as well as exposition of peripheral blood mononuclear cells (PBMCs) to vitamin D ex vivo, led to downregulation of TLRs’ expression and decrease of pro-inflammatory cytokine production. These findings have potentially significant implications for the treatment of a variety of conditions, where achieving optimal vitamin D levels may help reduce inflammation [199,200,201,202,203,204,205].

### 3.2. Toll-Like Receptors and Vitamin D in Cardiovascular Diseases

As was presented previously, both TLRs and vitamin D play an important role in a wide spectrum of cardiovascular diseases. A possible therapeutic approach may be treatment with specific TLR antagonists, e.g., Eritoran, Tak242, statins (fluvastatin) and angiotensin receptor blockers (candesartan) [19,206,207,208,209]. However, the easiest and most cost efficient way to reduce the pro-inflammatory effect of TLRs seems to be vitamin D supplementation. Alternatively, adequate vitamin 25(OH)D concentrations can also be obtained through dietary vitamin D intake and skin exposure to solar UVB radiation, although not as effective as supplementation. The recommended daily oral doses for infants and the general population beyond infancy are 400 IU and 800 IU, respectively. The upper tolerable intake level is considered to be 1000–4000 IU. For adults with hypovitaminosis D, the Endocrine Society recommends a daily vitamin D dose of 1500 to 2000 IU. It has been estimated that, for every 100 IU of vitamin D ingested, the blood level of 25(OH)D increases by 1 ng/mL. Daily doses of up to 10,000 IU are considered safe [104,210].

Randomized controlled trials concerning the therapeutic effects of vitamin D in cardiovascular diseases were not as appreciative as the observational studies due to the abovementioned reasons. Nevertheless, checking the vitamin D levels in all patients with cardiovascular disease and supplementation, if necessary, may be only beneficial.

### 3.3. Emerging Role of Vitamin K2 in Cardiovascular Diseases and Its Relationship with TLRs and Vitamin D

The latest research concerning this topic describes the emerging role of vitamin K2 (menaquinone) (especially subtypes MK-7, MK-8 and MK-9) in reducing cardiovascular risk. Vitamin K not only plays a vital role in blood coagulation, but also regulates tissue calcification, cell growth, bone formation and apoptosis. Vitamin K is needed for the carboxylation of proteins such as osteocalcin and matrix Gla protein, while vitamin D promotes their production. It leads to bone mineralization and inhibition of soft tissue calcification. As a result, optimal concentrations of vitamins D and K lower the risk of fractures and coronary heart disease [211,212,213,214,215,216,217,218]. To date, two intervention studies in healthy participants investigating the combined effect of vitamins D and K on vascular function and calcification have been conducted. The outcomes revealed maintained vessel wall characteristics of the carotid artery and less coronary artery calcium progression in the groups receiving dual supplementation [219,220]. Data concerning the relationship between TLRs and vitamin K are very scarce and no paper discussed the interplay among TLRs, and vitamins D and K so far. Nevertheless one study disclosed that menaquinone can suppress the expression of TLR2 and TLR4 as well as inhibit calcification of aortic intima and smooth muscle cells in *ApoE*^−/−^ mice [221]. Moreover, in vitro pretreatment of human monocyte-derived macrophages with menaquinone-7 for 30 h and subsequent activation with TLR agonists resulted in inhibition of pro-inflammatory cytokine production [222]. Abovementioned outcomes allow concluding that vitamin D and K2 have a synergistic effect and dual supplementation may provide an added benefit for patients’ bone and cardiovascular health.

### 3.4. Summary

To gain a deeper understanding of the role of TLRs and vitamin D in cardiovascular diseases, further experimental investigations as well as randomized controlled trials, most importantly with unified optimal 25(OH)D concentrations, are needed. It is significant not only in the treatment of individual patients, but also in prophylaxis, which may change the high mortality rates due to cardiovascular diseases currently observed.

## Abbreviations

TLRs	toll-like receptors
PRR	pattern-recognition receptor
PAMP	pathogen-associated molecular patterns
DAMP	danger-associated molecular patterns
TRIF	TIR-domain-containing adaptor inducing interferon-β
TIRAP	TIR domain-containing adaptor protein
TRAM	TRIF-related adaptor molecule
NF-κB	nuclear factor κ-light-chain-enhancer of activated B cells
HMGB1	high-mobility group box 1
CD	cluster of differentiation
Treg	T regulatory cell
DC	dendritic cell
Teff	T effector cell
MCP-1	monocyte chemotactic protein-1
TGF-β	transforming growth factor β
*ApoE*^−/−^ mice	apolipoprotein E knockout mice
iNOS	inducible nitric oxide synthase
ROS	reactive oxygen species
BMP-2	bone morphogenetic protein-2
Runx2	Runt-related transcription factor 2
TNF-*α*	tumor necrosis factor alpha
IL-6	interleukin 6
Ito	potassium current
IFN3	interferon regulatory factor 3
SHR	spontaneously hypertensive rat
IRAK1	interleukin-1 receptor-associated kinase 1
LPS	Lipopolysaccharide
TNF	tumor necrosis factor
IL-1β	interleukin 1β
NO	nitric oxide
NOS2	nitric oxide synthase 2
EMCV	encephalomyocarditis virus
CVB3	coxsackievirus group B serotype 3
UVB radiation	ultraviolet B radiation
25(OH)D	25-hydroxyvitamin D, calcidiol
1,25(OH)(2)D	1,25-dihydroxyvitamin D, calcitriol
VDR	vitamin D receptor
HREs	hormone response elements
NHANES	national health and nutrition examination survey
RAAS	renin-angiotensin system
NADPH	reduced nicotinamide adenine dinucleotide phosphate
eNOS	nitric oxide synthase
RANTES	regulated on activation, normal T cell expressed, and secreted
ICAM-1	intercellular adhesion molecule-1
PECAM-1	platelet-endothelial cell adhesion molecule-1
VCAM-1	vascular cell adhesion molecule-1
RAGE	receptor of advanced glycation end products
COX-2	cyclooxygenase-2
15PGDH	15-hydroxyprostaglandin dehydrogenase
EDCFs	endothelium-derived contracting factors
COX-1	cyclooxygenase-1
STEMI	ST-elevation myocardial infarction
NSTEMI	non-ST-elevation myocardial infarction
CHF	congestive heart failure
iPTH	intact parathyroid hormone
AF	atrial fibrillation
CABG	coronary artery bypass surgery
POAF	postoperative atrial fibrillation
PVC	premature ventricular complex
PTH	parathyroid hormone
DBP	vitamin D binding-protein
PBMC	peripheral blood mononuclear cell
